# Molecular Brightness Approach for FRET Analysis of Donor-Linker-Acceptor Constructs at the Single Molecule Level: A Concept

**DOI:** 10.3389/fmolb.2021.730394

**Published:** 2021-09-14

**Authors:** Taryn M. Kay, Cody P. Aplin, Rowan Simonet, Julie Beenken, Robert C. Miller, Christin Libal, Arnold J. Boersma, Erin D. Sheets, Ahmed A. Heikal

**Affiliations:** ^1^ Department of Physics and Astronomy, University of Minnesota Duluth, Duluth, MN, United States; ^2^ Department of Chemistry and Biochemistry, University of Minnesota Duluth, Duluth, MN, United States; ^3^ DWI-Leibniz Institute for Interactive Materials, Aachen, Germany

**Keywords:** FRET, FCS, donor-linker-acceptor, mTurquoise2.1, mCitrine, crTC2.1, molecular brightness, single molecule

## Abstract

In this report, we have developed a simple approach using single-detector fluorescence autocorrelation spectroscopy (FCS) to investigate the Förster resonance energy transfer (FRET) of genetically encoded, freely diffusing crTC2.1 (mTurquoise2.1–linker–mCitrine) at the single molecule level. We hypothesize that the molecular brightness of the freely diffusing donor (mTurquoise2.1) in the presence of the acceptor (mCitrine) is lower than that of the donor alone due to FRET. To test this hypothesis, the fluorescence fluctuation signal and number of molecules of freely diffusing construct were measured using FCS to calculate the molecular brightness of the donor, excited at 405 nm and detected at 475/50 nm, in the presence and absence of the acceptor. Our results indicate that the molecular brightness of cleaved crTC2.1 in a buffer is larger than that of the intact counterpart under 405-nm excitation. The energy transfer efficiency at the single molecule level is larger and more spread in values as compared with the ensemble-averaging time-resolved fluorescence measurements. In contrast, the molecular brightness of the intact crTC2.1, under 488 nm excitation of the acceptor (531/40 nm detection), is the same or slightly larger than that of the cleaved counterpart. These FCS-FRET measurements on freely diffusing donor-acceptor pairs are independent of the precise time constants associated with autocorrelation curves due to the presence of potential photophysical processes. Ultimately, when used in living cells, the proposed approach would only require a low expression level of these genetically encoded constructs, helping to limit potential interference with the cell machinery.

## Introduction

Förster resonance energy transfer (FRET) is a phenomenon in which energy is transferred, non-radiatively, from an excited donor (D) molecule to an acceptor (A) molecule that is in close proximity (≤10 nm) (Förster, 1948). FRET is often referred to as a “molecular ruler” for a wide range of applications due to its ability to measure intermolecular donor-acceptor distances (Stryer and Haugland, 1967; Jareserijman and Jovin, 2006; Lakowicz, 2006; Gadella, 2009; Choi et al., 2012). The energy transfer efficiency among a given donor-acceptor pair is dependent on the distance that separates the donor and acceptor molecule, the orientation parameter [κ (Jareserijman and Jovin, 2006)] of their relative dipole moments, and the spectral overlap between the donor’s emission and the acceptor’s absorption (Piston and Kremers, 2007; Gadella, 2009; Choi et al., 2012; Currie et al., 2017). In addition, FRET has been used successfully to study intermolecular interactions and their dynamics in a myriad of biological systems, both *in vitro* and *in vivo* (Lakowicz, 2006; Piston and Kremers, 2007; Zhang et al., 2013; Shrestha et al., 2015; Okamoto and Sako, 2017; Schwarz et al., 2019; Miller et al., 2020). FRET applications in scientific research include molecule-molecule interactions (Margineanu et al., 2016), conformational changes of biomolecules (Ha et al., 1996), and environmental sensing (Leopold et al., 2019; Schwarz et al., 2019; Miller et al., 2020).

Steady-state spectroscopy can be used to measure the energy transfer efficiency of FRET pairs based on the comparison of the time-averaged fluorescence intensity of the donor in the presence and absence of an acceptor (Lakowicz, 2006). This method has been applied to various mechanistic studies such as protein denaturation, enzyme-substrate binding, dye-DNA interaction, and RNA binding (Deniz et al., 2000; Zhuang et al., 2000; Elangovan et al., 2003; Ranjit et al., 2009; Zhang et al., 2013). Steady-state approaches for FRET analysis are also compatible with multi-channel confocal microscopy for investigating intermolecular interactions in living cells (Zhuang et al., 2000; Elangovan et al., 2003; Davey et al., 2008). The challenge with this approach, however, is the spectral overlap between the donor and acceptor that complicate the FRET analysis due to the potential of direct excitation of the acceptor, which results in overestimation of the FRET efficiency and therefore the donor-acceptor distance (Wu and Brand, 1994; dosRemedios and Moens, 1995; Ranjit et al., 2009).

Time-resolved fluorescence methods have also been used to investigate the excited-state dynamics of the donor in the presence and absence of the acceptor (Ranjit et al., 2009; Okamoto and Sako, 2017; Schwarz et al., 2019). The advantages of time-resolved fluorescence for FRET analysis include quantitative assessment of the excited state dynamics of the donor even in the presence of spectral overlap with the acceptor. Recently, for example, a family of mCerulean3–linker–mCitrine constructs that can be genetically encoded in living cells have been developed as environmental biosensors for *in vivo* studies of macromolecular crowding or ionic strength (Liu et al., 2017a; Liu et al., 2017b). In these constructs, the donor (mCerluean3) and acceptor (mCitrine) act as a FRET pair that are tethered together by either neutral (crowding) or two oppositely charged α-helices (ionic strength) in the linker region (Liu et al., 2017b). These environmental sensors have been investigated using ensemble averaging techniques such as time-resolved fluorescence (Currie et al., 2017; Schwarz et al., 2019; Miller et al., 2020) and time-resolved anisotropy (Currie et al., 2017; Leopold et al., 2019; Aplin et al., 2021) measurements. However, time-resolved fluorescence methods require expensive and specialized equipment as well as sophisticated users for experimental design and data analysis. Importantly, both steady-state spectroscopy and time-resolved fluorescence measurements suffer from averaging over large ensembles, which can wash out single molecular events (e.g., molecular or conformational states) that are key for kinetics and mechanistic studies (Remaut et al., 2005; Brunger et al., 2011; Choi et al., 2012). These traditional approaches can require relatively high expression levels of these genetically encoded constructs for FRET studies in living cells, which introduce the possibility of interfering with the biological cell machinery. In addition, high expression may lead to intermolecular FRET or maturation artifacts.

Genuine single molecule FRET (smFRET) studies have been reported on a wide range of immobilized biomolecules on surfaces (Roy et al., 2008; Lamichhane et al., 2010; Choi et al., 2012; Lerner et al., 2018; Qiao et al., 2021). In smFRET studies, the biomolecules of interest are labeled strategically using fluorescent dye molecules as donor-acceptor pairs and immobilized on a glass substrate. Both the fluorescence fluctuations of the donor and acceptor are recorded simultaneously. The corresponding FRET efficiency is then calculated during the time-dependent fluorescence fluctuation acquisition as a means for monitoring conformational changes of the biomolecule of interest (Roy et al., 2008; Lamichhane et al., 2010; Choi et al., 2012; Lerner et al., 2018; Qiao et al., 2021). Some of the challenges with this approach include the site-specificity of extrinsic dye labeling, potential surface immobilization effect on the conformational dynamics, the relative insensitivity to incomplete labeling, photobleaching and the limited signal-to-noise ratio, and low temporal resolution due to the low frame rate of the CCD camera (Roy et al., 2008).

Different modalities of fluorescence correlation spectroscopy (FCS), a single-molecule approach, have been used for non-invasive applications to study translational diffusion, chemical kinetics, and photophysical processes such as intersystem crossing to triplet electronic states and fluorescence blinking in fluorescent proteins (Widengren et al., 2001; Lakowicz, 2006; Haustein and Schwille, 2007; Elson, 2011; Sahoo and Schwille, 2011). Fluorescence dual-color cross-correlation spectroscopy (FCCS), for example, extends the temporal resolution to 10–100 nanoseconds by overcoming the inherent after-pulsing in the avalanche photodiode using the traditional, single-detector FCS. Dual color FRET-FCCS (Widengren et al., 2001; Torres and Levitus, 2007; Gurunathan and Levitus, 2008; Gurunathan and Levitus, 2010; Felekyan et al., 2012) has been applied to study conformational changes in RNA and proteins, DNA packaging, oligonucleotides, as well as oligomers of amyloid β-peptide (Remaut et al., 2005; Price et al., 2010; Price et al., 2011). The challenges with dual-color FCCS approach, however, include different excitation rates of the donor and acceptor, the difference in the fluorescence quantum yield, direct excitation of the acceptor under the donor’s excitation wavelength, accounting for the molecular brightness of the donor and acceptor under given experimental conditions, potential difference in the two channels detection efficiencies, long data acquisition time, photobleaching, and crosstalk between the donor-acceptor channels (Widengren et al., 2001; Sahoo and Schwille, 2011).

**FIGURE 1:**
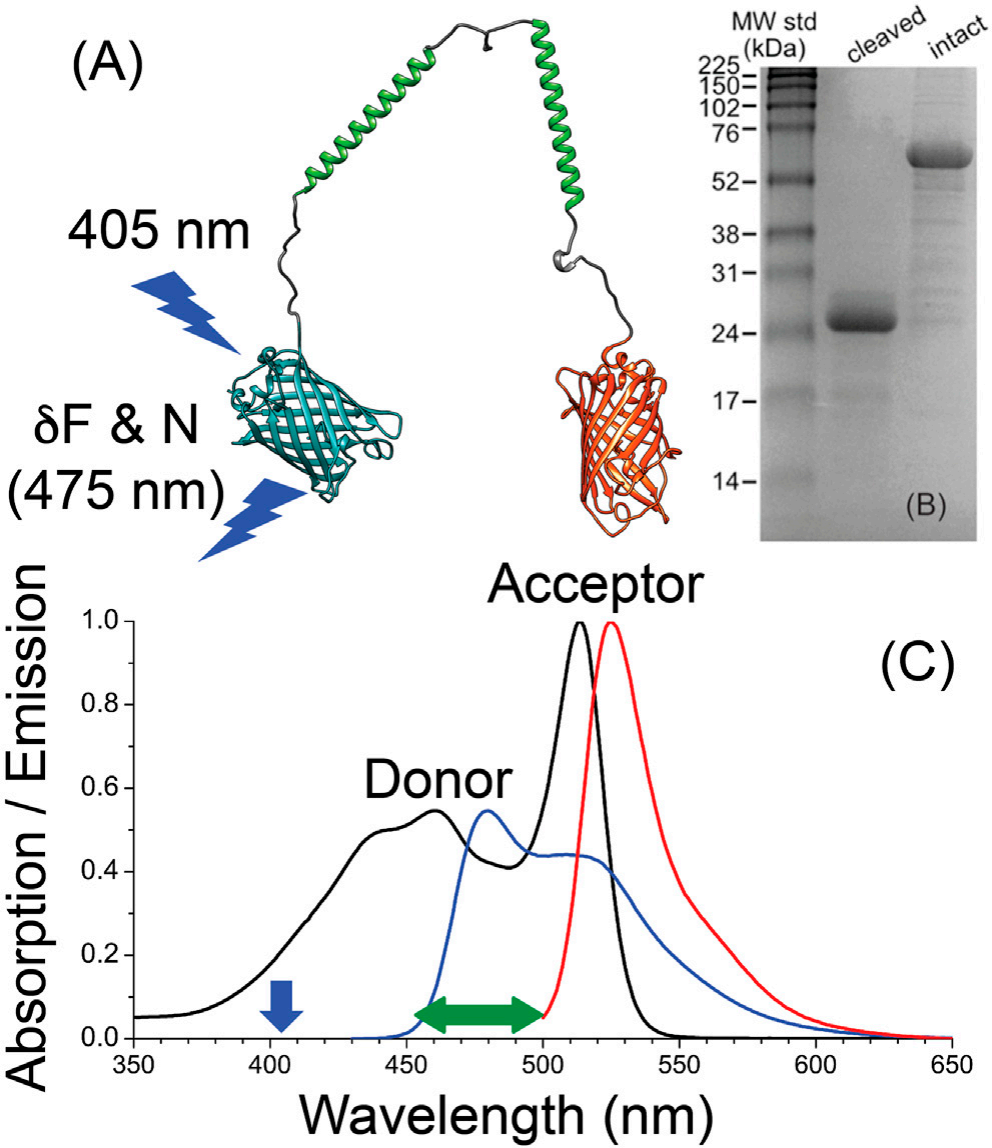
A schematic structure, SDS-PAGE gel, and steady-state spectroscopy of crTC2.1 construct (mTurquoise2.1-linker-mCitrine) (Liu et al., 2018) **(A)** The structure of crTC2.1 (mTurquoise2.1-linker-mCitrine), where the amino acid sequence of the linker region is –(GSG)_6_A (EAAAK)_6_A (GSG)_6_A (EAAAK)_6_A (GSG)_6_– **(B)** SDS-PAGE of the intact and cleaved construct, which shows the corresponding molecular weight and the efficiency of enzymatic cleavage reaction **(C)** The absorption and emission spectra of mTurquoise2.1-linker-mCitrine in a buffer, where the excitation (vertical arrow) and detection (horizontal arrow) of the donor in these FCS studies are shown.

In this report, we highlight a simple concept of using the fluorescence fluctuations and molecular brightness (number of emitted photon per molecule) for FRET analysis of a donor-linker-acceptor model construct at the single-molecule level using a traditional, single-detector FCS setup. As a proof of concept, we used crTC2.1 construct (mTurquoise2.1–linker–mCitrine) (Liu et al., 2018) as a model system (Figure 1A) for FCS studies under 405–nm excitation of the donor (mTurquoise2.1) in the presence and absence of the acceptor (mCitrine). The molecular brightness of the donor, both in the presence and absence of the acceptor, is calculated using the measured fluorescence fluctuation and number of molecules using the initial amplitude of the corresponding autocorrelation curve. Control experiments were also conducted under 488-nm excitation of the acceptor where FRET is unlikely to occur. In contrast with FRET-FCCS approaches (Widengren et al., 2001; Gurunathan and Levitus, 2008; Gurunathan and Levitus, 2010; Felekyan et al., 2012), the proposed concept relies on a traditional, single-detector FCS setup, which only requires analysis of the donor’s fluorescence fluctuations in the presence and absence of the acceptor. In the proposed experimental design, enzymatic cleavage of the linker region is required as a control on the donor alone under the same experimental conditions, which rule out the role of photophysical processes other than FRET in our analysis. Since the time scale of FRET is on the order of 1–10 nanoseconds and therefore beyond the temporal resolution of our experimental approach, the detailed nature of the autocorrelation decay model is not relevant to the molecular brightness based FRET analysis reported here.

## Donor’s Molecular Brightness for FCS-FRET Analysis: A Concept

The molecular brightness (the average number of fluorescence photons per molecule during their residence time in the observation volume) of the donor in the presence and absence of an acceptor can be used for FRET analysis of D-L-A constructs at the single-molecule level. We hypothesize that the molecular brightness of the donor alone is larger than that in the presence of an acceptor at close proximity. In this concept (Figure 1A), the fluorescence fluctuation and number of molecules using the initial amplitude of the autocorrelation function of the donor, in the presence and absence of the acceptor, are measured under 405 nm excitation and 475 nm detection (Figure 1C) as described below.

Using steady-state spectroscopy, the energy transfer efficiency in donor-acceptor FRET pairs is determined using the time-averaged fluorescence signal of the donor in the presence ($F_{DA}$) and absence ($F_{D}$) of the acceptor such that(Lakowicz, 2006)

$$E\left(\%\right)=\left(1-\frac{F_{DA}}{F_{D}}\right)\times 100$$ (1)

Generally, however, fluorescence intensity (*F*) of an ensemble of fluorophores, excited by light intensity (I), is given by(Xu and Webb, 1996)

F=φησCI (2)

Where φ is the fluorescence quantum yield of the fluorophore, η is the detection efficiency of the fluorescence signal that is dependent on the experimental setup, σ is the absorption cross-section of the fluorophore at a given excitation wavelength, and *C* is the concentration of the fluorophore in the sample under investigation. At the single-molecule level, the concentration (*C*) of the fluorophore can be related to the number of molecules (*N*) in a given volume (*V*) using Avogadro’s number ($N_{A}$) such that Eq. 2 can be rewritten as:

$$F=\frac{\varphi \eta \sigma I}{VN_{A}}\times N$$ (3)

In a traditional FCS experiment, the observation volume (*V*) under 488 nm illumination can be determined using a photostable fluorphore (rhodamine-110) with known diffusion coefficient as a reference (Currie et al., 2017; Lee et al., 2018; Aplin et al., 2020). In order to avoid minor differences in the concentrations of prepared samples, the fluorescence intensity (F) can be rewritten in terms of the molecular brightness (ψ) such that:

$$\psi =\frac{F}{N}=\frac{\varphi \eta \sigma I}{VN_{A}}$$ (4)

The molecular brightness is defined as the average number of fluorescence photons detected per fluorophore during their random walk in the observation volume. Under the same experimental conditions of laser intensity, excitation wavelength of the donor, observation volume, and the detection efficiency, the FRET efficiency in Eq. 1 can be rewritten in terms of the molecular brightness of the donor in the presence ($\psi_{DA}$) and absence ($\psi_{D}$) of the acceptor such that:

$$E\left(\%\right)=\left(1-\frac{\psi_{DA}}{\psi_{D}}\right)\times 100$$ (5)

As a result, the FRET analysis can be carried out on freely diffusing donor–linker–acceptor construct at the single molecule level in terms of the molecular brightness of the excited donor, in the presence and absence of an acceptor, using traditional, single-detector FCS setup where the fluorescence fluctuation and number of molecules can be measured.

## Materials and Experimental Design Using Conventional FCS Setup

### Molecular Model System

As a proof of concept, we carried out traditional FCS experiments on crTC2.1 construct (mTurquoise2.1–linker–mCitrine) (Liu et al., 2018) as a model donor–linker–acceptor (D-L-A) system (Figure 1A) under 405 nm excitation of the donor (mTurquoise2.1) in the presence and absence of the acceptor (mCitrine). The amino acid sequence of the linker region in this crTC2.1 construct is –(GSG)_6_A (EAAAK)_6_A (GSG)_6_A (EAAAK)_6_A (GSG)_6_– (Liu et al., 2018). This model system was selected due to its low energy transfer efficiency (7.1 ± 0.5)% as measured using time-resolved fluorescence (Aplin et al., 2020) to test the sensitivity of the proposed FCS approach. For the control experiments on the donor alone, the linker region of the FRET probe (mTurquoise2.1–link–mCitrine) was digested using the serine protease, proteinase K (Leopold et al., 2019; Schwarz et al., 2019; Miller et al., 2020; Aplin et al., 2021) (Figure 1B). The absorption and emission spectra of crTC2.1 construct in a buffer are also shown (Figure 1C), where the excitation wavelength of the donor (405 nm) and its fluorescence detection can be visualized (arrows). The fluorescence peaks of both the donor (480 nm) and acceptor (525 nm) are normalized with the corresponding absorption bands (460 and 514 nm, respectively). The absorption cross-section of mTurquoise2.1 is larger than that of mCerulean3 (not shown) relative to that of mCitrine. Importantly, the spectral overlap between mTurquoise2.1 emission and the mCitrine absorption is slightly larger than that of mCerulean3, which in principle would enhance the FRET efficiency. Unfortunately, however, the fluorescence of mTurquoise2.1 exhibits larger overlap with the mCitrine’s emission. Rhodamine–110 in nanopure water was used as a reference with known diffusion coefficient to calibrate the FCS setup under 488-nm excitation of the acceptor.

### FCS Setup

A home-built fluorescence correlation spectroscopy (FCS) setup has been described in detail elsewhere (Currie et al., 2017; Lee et al., 2018). Briefly, a collimated continuous wave (cw) laser was generated using a diode laser (405 nm, PhoxX405-60, Omicron-Laserage Laserprodukte GmbH, Germany) for exciting the donor in the presence and absence of the acceptor. Another 488-nm laser beam (Coherent Sapphire 488–20) was also used to excite the acceptor for control experiments. The laser beam (405 or 488 nm) was then conditioned and steered towards an inverted microscope (IX81, Olympus) via the back-exit port and through the microscope objective (1.2NA, water immersion, infinity corrected, 60x, Olympus) for both sample excitation and emission detection. The average power of the laser at the sample used in these experiments ranged between 7.3 μW and 13.6 μW (at 405 nm) with estimated laser intensities at the sample of 55.4 kW/cm^2^ and 103.9 kW/cm^2^, respectively. Under 488 nm excitation of the acceptor, however, the average power of the laser was about 2 μW with laser intensity at the sample of 1.6 kW/cm^2^. A dichroic mirror (395DM for 405 nm excitation or 490DM for 488 nm excitation) was used to separate the fluorescence emission from the excitation laser. The collimated epifluorescence signal was first filtered (using a bandwidth filter, 475/50 nm for donor detection or 531/40 for acceptor detection) and then focused on a confocal pinhole (an optical fiber with 50 μm diameter). The fluorescence signal was then detected by an avalanche photodiode (APD, SPCM CD-2969, PerkinElmer, Fremont, CA), amplified, and correlated using an external multiple-tau-digital correlator (ALV/6010–160, Langen/Hessen, Germany).

### FCS Data Analysis

In a single-detector FCS, the time-dependent fluorescence fluctuations, δF(t), are recorded as single molecules diffuse through an open observation volume such that (Tian et al., 2011)

δF(t)=F(t)−〈F(t)〉 (6)

The observed fluorescence fluctuation is caused by the fluctuation of the concentration (1–50 nM) of molecules in the observation volume due to translational diffusion, chemical reactions, and fast photophysical processes such as fluorescence blinking in fluorescent proteins, conformational changes of biomolecules, or intersystem crossing (Elson and Magde, 1974; Magde et al., 1974; Widengren and Rigler, 1997). The corresponding autocorrelation curve, G(τ), is calculated using the measured time-dependent fluorescence fluctuation, δF(t), as a function of a lag time (τ), *i.e.,* δF(t+τ), such that (Schwille, 2001; Elson, 2011)

$$G\left(\tau \right)=\frac{\langle \delta F\left(t\right)⊗\delta F\left(t+\tau \right)\rangle }{{\langle F\left(t\right)\rangle }^{2}}$$ (7)

Assuming a three-dimensional (3D) Gaussian profile of the observation volume, the fluorescence fluctuation autocorrelation curve, $G_{D}\left(\tau \right)$, due to translational diffusion and a fast photophysical process (with a time scale of $\tau_{f}$) can be written as following (Huang et al., 2002)

$$G\left(\tau \right)=\frac{1}{N}{\left(1-\frac{\tau }{\tau_{D}}\right)}^{-1}{\left(1-\frac{\tau }{s^{2}\tau_{D}}\right)}^{-1/2}\times \left\{1-A_{f}+A_{f}.e^{-t/\tau_{f}}\right\}$$ (8)

Here, *N* is the average number of molecules residing in the observation volume, whose structure parameter (*s*) is the ratio of the axial (*z*) to the lateral (*r*) extension of that volume such that s=z/r. The autocorrelation curve depends also on the population fraction ($A_{f}$) of the molecules undergoing a fast photophysical process. According to Eq. 8, as the number of molecules in the observation volume increases, the initial amplitude of the autocorrelation curves will decrease. In addition, lower cw laser intensity (7.3–13.6 μW at 405 nm) of the donor was used, where the fluorescence signal was linear with laser intensity. Within this linear regime, the time-dependent photobleaching of the fluorescence signal was negligible. Low cw laser illumination in FCS experiments also minimizes direct excitation of the acceptor.

The measured diffusion time ($\tau_{D}$) and the diffusion coefficient (*D*) of a given molecule are related, where:

$$\tau_{D}=\frac{r^{2}}{4D}$$ (9)

To calibrate FCS setup under 488 nm excitation, the diffusion time of a reference fluorophore (e.g., rhodamine 110) with a known diffusion coefficient is used to determine the radius of the observation volume (Currie et al., 2017; Lee et al., 2018; Aplin et al., 2020).

According to Eq. 8, the initial amplitude of the autocorrelation function, $G_{i}\left(\tau =0\right)$, of the *i*th species (*i* = *D* for cleaved*, i* = *DA for intact*) equals the inverse of the average number of molecules ($N_{i}$) residing in the open observation volume such that:

$$G_{i}\left(\tau =0\right)=\frac{1}{N_{i}}$$ (10)

Using the time-averaged fluorescence signal, 〈δF(t)〉, and the corresponding number of molecules (*N*) in the observation volume, the molecular brightness (ψ) can be calculated experimentally using FCS such that:

$$\psi_{i}=\frac{\langle \delta F_{i}\left(t\right)\rangle }{N}$$ (11)

Where the molecular brightness of the donor in the absence and presence of the acceptor are measured under the same experimental conditions. Using the measured molecular brightness (Eq. 4 or 11), the corresponding energy transfer efficiency (Eq. 5) can be determined for donor–linker–acceptor at the single-molecule level in a given environment. In these FCS measurements, about 20 nM concentrations of crTC2.1 were used for fluorescence fluctuation analysis.

### Practical Guideline for Reliable Experimental Design

The measured time-dependent fluorescence fluctuations of the cleaved and intact constructs were measured under the same experimental conditions and then used to calculate the corresponding autocorrelation curves and the corresponding molecular brightness. Under a given laser intensity, up to 20 fluorescence fluctuation traces were measured at 5 or 10 s/trace and the average fluorescence signal (δF) per trace was automatically recorded. However, the excitation/detection conditions should be optimized to ensure stable (i.e., negligible photobleaching) fluorescence fluctuation signal and number of molecules during these data acquisition.

The corresponding autocorrelation curves were then analyzed using OriginPro8.0 software for each trace to determine both the number of molecules, G(τ=0)=1/N, under a given experimental condition and the corresponding average fluorescence fluctuation per trace. It is recommended that the average number of freely diffusing molecules should be less than 100 molecules residing in the observation volume. Under the same experimental conditions, the buffer alone (i.e., no donor–linker–acceptor construct) was measured and any background signal was then subtracted from the fluorescence fluctuation signal prior to calculating the molecular brightness associated with each trace. Since the molecular brightness of the donor and the donor–acceptor pair were measured under the same experimental conditions, the complexity associated with additional photophysical processes can be ruled out in the proposed experimental design (see below). For the same reasons, the effects of nonideal optical conditions of our inverted microscope under 405 nm excitation and the glass coverslip would be negligible. These measurements were repeated on different days of laser alignment, laser intensities, and sample preparations for reproducibility test.

## Results and Discussion

### Fluorescence Fluctuation Analysis of the crTC2.1 Construct Under 405-nm Excitation Using Traditional FCS

We hypothesize that the molecular brightness of the cleaved donor–linker–acceptor construct is larger than that of the intact counterpart under 405 nm excitation and 475/50 detection of the donor’s emission. To test this hypothesis, we carried out fluorescence fluctuation analysis using a traditional, single detector FCS on crTC2.1 construct in a 10 mM sodium phosphate (NaPi) buffer (pH 7.4) at room temperature.

In these experiments, we measured time-dependent fluorescence fluctuations of the donor (mTurquoise2.1) in crTC2.1 construct both in the presence (i.e., intact) and absence (i.e., enzymatically cleaved) of the acceptor (mCitrine). The measured time-dependent fluorescence fluctuation traces of the donor were then used to calculate the corresponding autocorrelation curve for each trace, where the corresponding number of molecules was determined and used for molecular brightness per trace prior to statistical analysis. This experimental design helps to eliminate the possible effects of fluorescence blinking on the estimated FRET analysis under 405 nm excitation.

**FIGURE 2:**
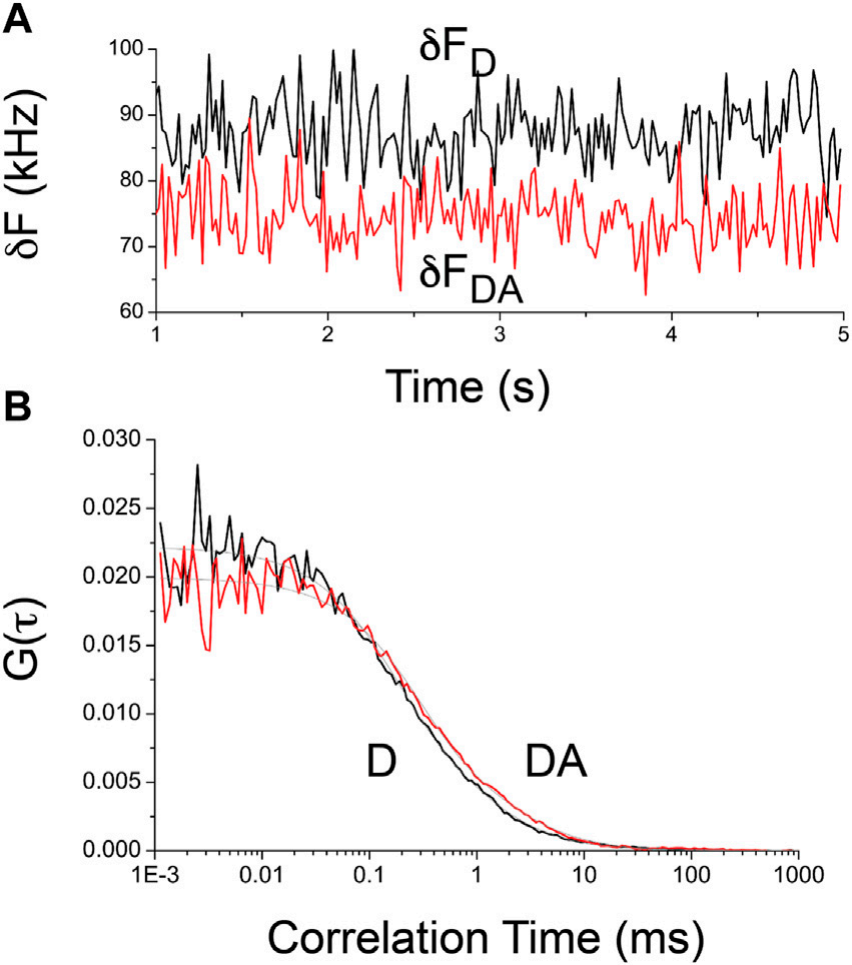
Representative time-dependent fluorescence fluctuations and autocorrelation curves of intact and enzymatically-cleaved crTC2.1 construct **(A)** Representative fluorescence fluctuation traces of cleaved (black trace) and intact (red trace) of crTC2.1 indicate difference in the average number of fluorescence photons detected during their translational diffusion through the open observation volume **(B)** The corresponding autocorrelation curves of cleaved (black curve) and intact (red curve) of crTC2.1. Under 405 nm excitation (7 μW), the fitting parameters of these representative autocorrelation curves are as following: the translational diffusion times are 0.57 ms (cleaved) and 1.0 ms (intact) with a fast photophysical process with time scales of 180 μs (cleaved, 41%) and 299 μs (intact, 47%). The corresponding number of molecules residing in the observation volume here are 45 (cleaved) and 50 (intact).

Figure 2 shows a representative fluorescence fluctuation of the donor in the presence and absence of the acceptor (Figure 2A) as well as the corresponding autocorrelation curves used for determining the number of molecules residing in the observation volume (Figure 2B). These representative fluorescence fluctuation traces (Figure 2A) and the corresponding autocorrelation curve (Figure 2B) of the donor alone exhibit a smaller number of molecules and larger average fluorescence fluctuation signal than the intact counterpart. The corresponding fitting parameters of these representative autocorrelation curves (see Figure 2 caption) indicate that the diffusion time of the intact crTC2.1 (∼65 kDa, 1.0 ms) is larger than the cleaved counterpart (∼26 kDa, 0.45 ms) due to the different molecular weights (Figure 1B). The observed difference in the fluorescence signal of the donor in the presence and absence of the acceptor (Figure 2) can be attributed to a slight difference in either the prepared concentrations or FRET efficiency. The molecular brightness in this proposed concept rules out the possible difference in the prepared sample concentrations used in these FCS–FRET analyses.

**FIGURE 3:**
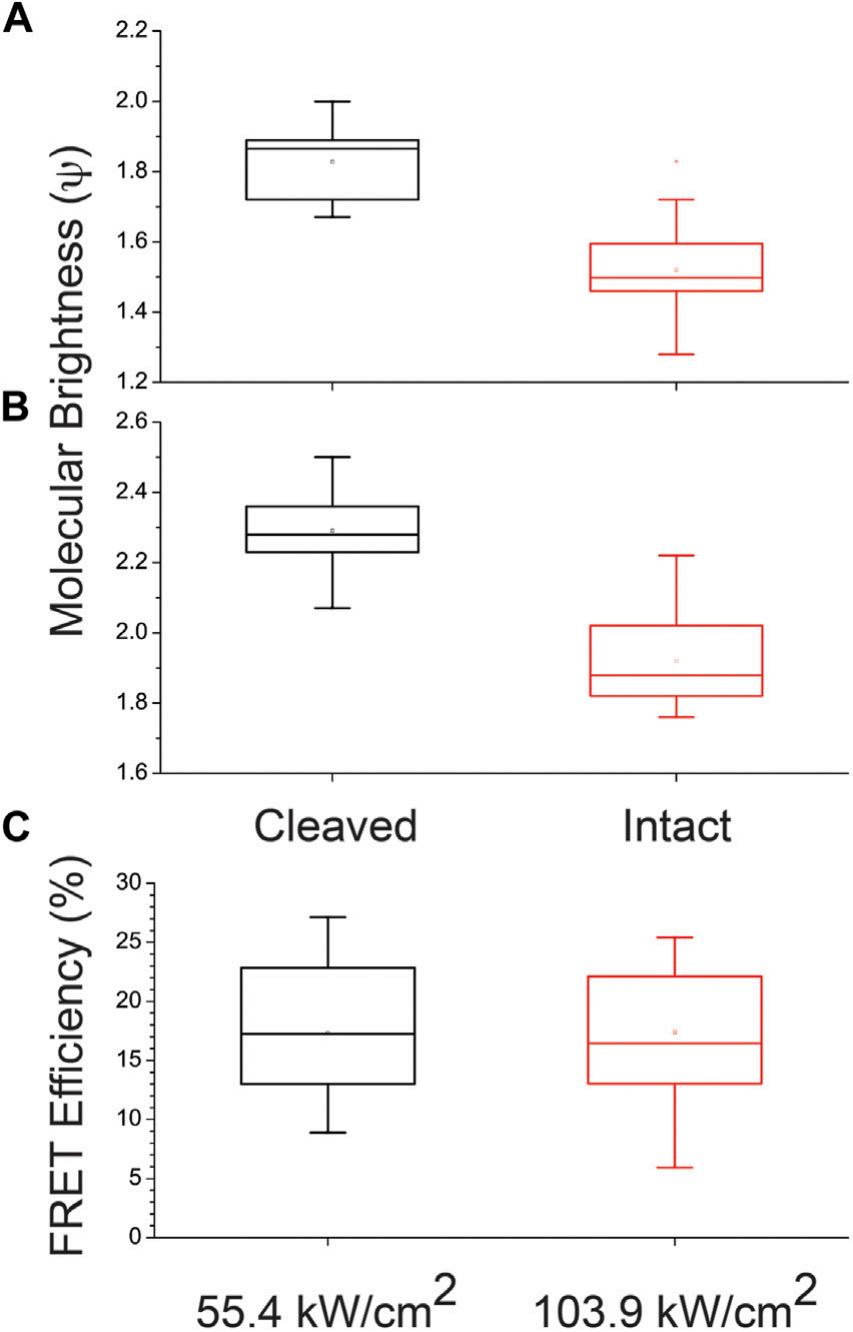
Representative box and whisker plots of the molecular brightness and energy transfer of crTC2.1 (mTurquoise2.1-linker-mCitrine) at the single-molecule level **(A)** The molecular brightness (ψ, kHz/molecule) of intact and enzymatically cleaved crTC2.1 in NaPi buffer, measured under 405-nm excitation (7.3 μW or 55.4 kW/cm^2^) and 475/50 nm detection **(B)** The molecular brightness of intact and enzymatically cleaved crTC2.1 in NaPi buffer, measured under 405 nm excitation at slightly higher laser intensity (103.9 kW/cm^2^ or 13.6 μW) **(C)** The corresponding FRET efficiency of crTC2.1 as estimated using the molecular brightness of both intact and cleaved construct. The line running through each box is the median value for the range of data set (number of trials = 18) being plotted.

Figures 3A, B shows the molecular brightness of the intact and cleaved crTC2.1 under slightly different laser intensity (55.4 kW/cm^2^ *versus* 103.9 kW/cm^2^), within the linear regime of laser intensity dependence of the fluorescence signal. These box and whisker plots display the median as well as the upper and lower quartiles, which best reflect the range of the experimental data at the single-molecule level. For example, the molecular brightness of the intact construct (1.5 ± 0.1 kHz/molecule) is lower than that of the cleaved counterpart (1.8 ± 0.1 kHz/molecule) under the 405-nm excitation laser intensity (55.4 kW/cm^2^) as shown in Figure 3A. However, the molecular brightness of a given fluorophore increases as the excitation laser intensity increases (Figure 3B). For example, under a slightly higher laser intensity (103.9 kW/cm^2^), the molecular brightness of the intact construct (1.9 ± 0.1 kHz/molecule) is lower than that of the cleaved counterpart (2.3 ± 0.1 kHz/molecule) as shown in Figure 3B. These results support our stated hypothesis and the observed difference in the measured molecular brightness is attributed to FRET using the proposed experimental design.

### Traditional FCS-FRET Analysis of crTC2.1 Construct Under 405-nm Excitation of the Donor

Using the above measured molecular brightness of both cleaved and intact crTC2.1 construct (Figures 3A,B), we calculated the corresponding FRET efficiency of crTC2.1 as described in Eq. 5. As shown in Figure 3C, the estimated energy transfer efficiency is (17.3 ± 5.4)% at the single molecule level using 55.4 kW/cm^2^ excitation laser intensity. A statistically insignificant difference in the calculated FRET efficiency was observed (17.4 ± 5.6)% under 103.9 kW/cm^2^ excitation laser (Figure 3C) within this linear regime.

These single-molecule studies also yield a relatively larger FRET efficiency as compared with ensemble studies using time-resolved fluorescence, where (7.1 ± 0.5%) was measured on the same construct (Aplin et al., 2020). The statistical spreading of our molecular brightness and the estimated FRET efficiency could be attributed to the single-molecular processes or molecular conformations observed in FCS of freely diffusing crTC2.1 as compared with the traditional time-resolved fluorescence (Aplin et al., 2020). In addition, these single molecule studies yield a larger FRET efficiency as compared with the ensemble averaging, time-resolved fluorescence approach (Aplin et al., 2020). As a control for reproducibility and general applicability of the proposed approach, we carried out similar FCS measurements on GE (mCerulean3–linker–acceptor) construct, excited at 405 nm, and similar trends were observed (data not shown).

Taken together, our experimental data support our hypothesis as well as the proposed concept for FRET analysis at the single molecule level using the simple, conventional FCS setup, where the fluorescence fluctuation of the donor in both the intact and cleaved donor–linker–acceptor construct can be measured directly.

### Fluorescence Fluctuation Analysis of the Acceptor (No FRET) Under 488-nm Excitation: Additional Controls

**FIGURE 4:**
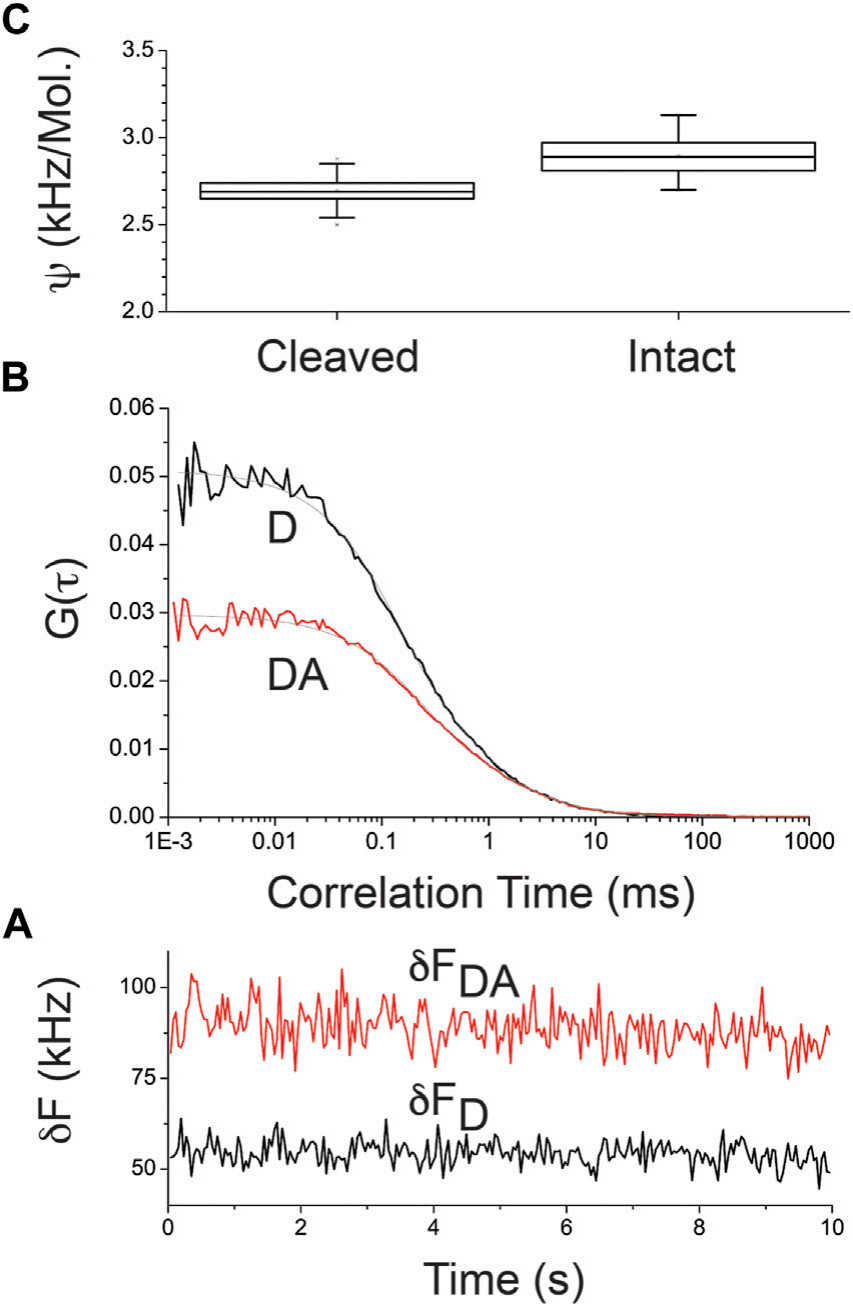
Representative autocorrelation curves and molecular brightness of cleaved and intact crTC2.1 construct under 488-nm excitation **(A)** Representative fluorescence fluctuation traces of cleaved (D, black curve) and intact (DA, red curve) crTC2.1, which were measured under the same experimental conditions **(B)** The corresponding autocorrelation curves of cleaved (D, black curve) and intact (DA, red curve) crTC2.1 under 488 nm excitation and 531/50 nm detection using these representative fluorescence fluctuations **(A) (C)** Representative box and whisker plots of the molecular brightness (ψ, kHz/molecule) of intact and enzymatically cleaved crTC2.1 in 10 mM NaPi buffer are also shown (*n* = 20). Under 488 nm excitation (2 μW or 1.6 kW/cm^2^) observation volume, the fitting parameters of these representative autocorrelation curves **(B)** are as following: the translational diffusion times are 0.47 ms (cleaved) and 0.83 ms (intact) with a fast photophysical process with time scales of 150 μs (cleaved, 44%) and 233 μs (intact, 44%). The corresponding number of molecules residing in the observation volume here are 20 (cleaved) and 34 (intact).

We hypothesize that the molecular brightness of the cleaved and intact donor–linker–acceptor construct is the same under 488 nm excitation and 531/40 detection of the acceptor. To test this hypothesis, we carried out complementary measurements on molecular brightness of cleaved and intact crTC2.1 construct under 488-nm excitation (laser power = 2 μW or intensity = 1.6 kW/cm^2^) of the acceptor (mCitrine), detected at 531/50 nm. Representative fluorescence fluctuations (Figure 4A) and the corresponding autocorrelation curves (Figure 4B) of cleaved and intact crTC2.1 are shown in Figure 4. The corresponding molecular brightness of crTC2.1 construct was also calculated over 20 fluorescence fluctuation traces (10 s/each) under the same experimental conditions (Figure 4C). Our results show that, in the absence of FRET at 488 nm excitation of the acceptor, the molecular brightness of the intact (2.9 ± 0.1 kHz/molecule) crTC2.1 is slightly larger than that of the cleaved counterpart (2.7 ± 0.1 kHz/molecule), which is in contrast with our observation under 405 nm excitation (Figure 4C). The slight increase in the molecular brightness of the intact construct under 488 nm excitation can be attributed to an enhanced spectral overlap between the donor and acceptor in crTC2.1 construct.

As an additional control, complementary measurements were carried out on GE (mCerulean3–linker–mCitrine) construct under 488 nm excitation, which reveal similar molecular brightness for both cleaved (2.7 ± 0.2 kHz/molecule) and intact (2.7 ± 0.2 kHz/molecule) construct (data not shown). The results on GE (mCerulean3–linker–mCitrine) construct support the general applicability of the proposed FCS-FRET approach.

It is worth mentioning that the measured translational diffusion times of cleaved and intact crTC2.1 (Figure 4A) are consistent with their molecular mass (Figure 1B) as reported previously under 488 nm excitation (Aplin et al., 2020). Under 488-nm excitation, the FCS observation volume was calibrated using rhodamine-110 with known diffusion time (Aplin et al., 2020).

## Conclusion

As a proof of concept, we have demonstrated a simple approach for FRET analysis of freely diffusing crTC2.1 construct, at the single molecule level, using a traditional, single detector FCS setup. In this approach, the donor in mTurquoise2.1–linker–mCitrine construct was excited at 405 nm and the fluorescence fluctuation of the donor (475/50 nm) in the presence and absence of the acceptor (mCitrine) are directly measured under the same experimental conditions of laser intensity and detection efficiency. The initial amplitude of the corresponding autocorrelation curve was used to calculate the average number of molecules residing in the observation volume. Then, we calculated the corresponding molecular brightness of both cleaved and intact crTC2.1 construct under the same experimental conditions to rule out any minor changes in the concentration of the prepared samples. These molecular brightness values were then used to determine the energy transfer efficiency in crTC2.1 construct under 405-nm excitation of the donor (mTurquoise2.1).

Our results support our stated hypothesis that the molecular brightness of the donor alone is larger than that of the intact mTurquoise2.1–linker–mCitrine construct, which is attributed to FRET. These single-molecule studies also yield a relatively larger FRET efficiency as compared with ensemble studies using time-resolved fluorescence on the same construct (Aplin et al., 2020). The statistical spreading of the observed molecular brightness and the estimated FRET efficiency could be attributed to the single-molecular processes or molecular conformations observed in FCS as compared with the traditional, ensemble-averaging, time-resolved fluorescence.

Control experiments on the intact and cleaved crTC2.1, under 488 nm excitation of the acceptor, yield a slightly larger molecular brightness of the intact crTC2.1 construct than the cleaved counterpart, which is in contrast to our 405-nm excitation results, due to the absence of FRET. Additional control studies were carried out on another GE (mCerulean3-linker-mCitrine) construct under 488-nm excitation of the acceptor, where the observed molecular brightness of both cleaved and intact construct were the same.

It is worth noting that intrinsically fluorescent proteins and their mutations are known to undergo both pH- and light-induced fluorescence blinking (Schwille et al., 1999; Heikal et al., 2000; Schwille et al., 2000; Hess et al., 2003; Hess et al., 2004). Light-induced blinking of mTurquoise2.1 in the hetero-FRET sensor may be present under 405-nm illumination and, in principle, should complicate the FRET analysis by overestimating the energy transfer efficiency. However, such blinking process is likely to be present in both the cleaved and intact sensor under the same conditions. As a result, it is reasonable to exclude the light-driven blinking as a source for the observed differences in the molecular brightness and therefore the estimated FRET efficiency.

The proposed FCS approach takes advantage of the traditional, single-detector FCS setup for selective excitation of the donor in the presence and absence of the acceptor for FRET analysis at the single-molecule level. Another key advantage here is that the proposed approach minimizes the ensemble averaging by at least three orders of magnitude and therefore interesting single molecule events or conformations will not be washed out. Finally, this FCS-FRET approach does not rely on the exact nature of the autocorrelation decay that could be complicated in the presence of a number of photophysical processes in any given donor–linker–acceptor construct.

The proposed concept would eliminate the need for high expression levels of these sensors when used in living cells while also eliminating the ensemble averaging that is inherent in other techniques. This FCS concept is generally applicable for FRET analysis in a wide range of donor–linker–acceptor constructs, where the donor and acceptor are fluorescent proteins linked together with amino acid sequence that can be enzymatically digested for control studies. This could be considered as a limitation of the proposed approach here, but that is also the same for any reliable time-resolved fluorescence measurements where control studies on the donor alone must be carried out under the same experimental conditions. To overcome this limitation in future studies in living cells, for example, it is possible to repeat the same site-specific FCS measurements on two cell cultures, one expressing donor–linker–acceptor construct only and another expressing the donor alone. Laser-induced quenching of the acceptor in living cells expressing donor–linker–acceptor construct may also work. For solution studies on untethered FRET pairs, it is also conceivable to vary the relative concentrations of the acceptor with respect to the donor while monitoring the molecular brightness of freely diffusing molecules using a traditional FCS setup.

## Data Availability

The original contributions presented in the study are included in the article/Supplementary Material, further inquiries can be directed to the corresponding author.
